# Spatiotemporal analysis of within-country imported malaria in Brazilian municipalities, 2004–2022

**DOI:** 10.1371/journal.pgph.0003452

**Published:** 2024-07-15

**Authors:** Nicholas J. Arisco, Cassio Peterka, Marcia C. Castro

**Affiliations:** 1 Department of Global Health and Population, Harvard TH Chan School of Public Health, Boston, Massachusetts, United States of America; 2 Department of Health and Environmental Surveillance, Ministry of Health, Brasília, Federal District, Brazil; CSIR-Indian Institute of Chemcial Technology, INDIA

## Abstract

Human mobility has challenged malaria elimination efforts and remains difficult to routinely track. In Brazil, administrative records from the Ministry of Health allow monitoring of mobility locally and internationally. Although most imported malaria cases are between municipalities in Brazil, detailed knowledge of patterns of mobility is limited. Here, we address this gap by quantifying and describing patterns of malaria-infected individuals across the Amazon. We used network analysis, spatial clustering, and linear models to quantify and characterize the movement of malaria cases in Brazil between 2004 and 2022. We identified sources and sinks of malaria within and between states. We found that between-state movement of cases has become proportionally more important than within-state, that source clusters persisted longer than sink clusters, that movement of cases into sinks was seasonal while movement out of sources was not, and that importation is an impediment for subnational elimination in many municipalities. We elucidate the vast travel networks of malaria infected individuals that characterize the Amazon region. Uncovering patterns of malaria case mobility is vital for effective microstratification within Brazil. Our results have implications for intervention stratification across Brazil in line with the country’s goal of malaria elimination by 2035.

## Introduction

Human mobility within and between countries historically has challenged malaria elimination efforts [1, 2]. Mobile, malaria-infected individuals can introduce parasites into regions where parasite prevalence, and thus local immunity, is very low, resulting in outbreaks of malaria, or in areas that had previously achieved elimination resulting in reintroduction of the disease [1, 3]. Furthermore, malaria vectors are extant in all parts of Brazil, which may pose challenges on the path to elimination with respect to receptivity and vulnerability of regions [4]. Tracking mobile populations can be challenging and migratory movements may happen unexpectedly (e.g., civil strife, natural disaster) making it difficult to forecast, surveil, and allocate resources for control [5–7]. Understanding patterns of malaria importation can improve surveillance and facilitate the planning and implementation of elimination programs [8–10]. Yet, malaria-endemic countries aiming at elimination have limited capacity to regularly track international and within-country malaria importation [11].

Malaria control efforts in Brazil have varied over time. Between 2000 and 2003, following a peak of 630,985 cases in 1999, Brazil launched the Intensification Plan of Malaria Control Activities in the Legal Amazon (PIACM) to reduce malaria cases and mortality by half in 2001 and 2002, respectively [12]. The success of this plan spurred the launch of the National Malaria Prevention and Control Program (NMCP) in 2003, which expanded its goals to include building local capacity and establishing a national surveillance program known as the Malaria Epidemiological Surveillance Information System (Sivep-Malaria) [13].

Control efforts contributed to reducing malaria incidence in the Amazon by more than 75% between 2000 and 2015, spurring the launch of a plan for the elimination of *P*. *falciparum* in November of 2015 [14]. In 2010, the Global Fund supported a project aimed at strengthening local public health capacity in the Amazon by ensuring early diagnosis, timely treatment, distribution of bed nets, and improving municipal and state-level management [15]. Reductions in cases continued, with 2016 marking the lowest recorded year for malaria case counts in Brazil in 35 years. Though the NMCP continued its efforts toward malaria elimination, case counts began increasing in 2017 [16]. In 2019 and 2020, cases plateaued and began to decline again, trending toward 2016 levels. Lastly, in 2022, a new elimination plan was launched to achieve zero malaria cases and deaths by 2035 [17].

Roughly 99.5% of cases in Brazil are currently reported in the Amazon region. Malaria transmission in the Amazon, defined as frontier malaria [18–20], operates at three levels: micro (through poor housing quality, intense vector exposure, and heightened vector density due to ecosystem modification), community (through high human mobility rates, disenfranchisement of at-risk individuals such as loosening of protections on indigenous areas, and weak healthcare institutions), and state/national levels (through sporadic, unplanned development for agricultural purposes or through planned settlement projects) [18]. The Amazon is marked by intense population mobility due to economic activity, exploitation of resources through forestry/mining, and interconnectedness of locations via road development and river networks [21–26]. In addition, the Brazilian Amazon shares a border with seven countries in South America, and thus cross-border malaria is an issue of concern. While our previous work has shown that 85% of imported malaria cases in Brazil happen within the country [11, 27], we lack knowledge regarding the spatial and temporal characteristics of in-country importation.

Through Sivep-Malaria, Brazil routinely collects information that allows for systematic monitoring of importation both locally and internationally [11]. Nevertheless, those data have not been thoroughly analyzed to inform surveillance and elimination efforts. The few studies that have analyzed importation of malaria cases are restricted to small areas, short periods, are outdated, and/or have coarse spatial scale [28–34]. Brazil’s new elimination plan, launched in 2022, aims to achieve zero malaria in four phases: (i) reduce malaria cases to less than 68 thousand by 2025, (ii) eliminate *Plasmodium falciparum* malaria and deaths due to malaria by 2030, (iii) achieve zero malaria cases and deaths by 2035, and (iv) prevent the reintroduction of malaria from 2035 onwards [17]. Yet, the systematic guidance for stratification and planning of interventions for malaria elimination accounting for malaria importation is hampered by limited knowledge of the dynamics of importation. Here we analyze the patterns of malaria importation in Brazil, identifying municipalities (N = 5,570) that are sources and sinks of malaria, considering 18 years of monthly data (from 2004, shortly after the National Malaria Prevention and Control Program, NMCP, was launched, through the end of 2022. We aim to offer context to mobility of malaria-infected individuals in Brazil by: (1) defining and quantifying the major types of movement of malaria infections occuring in Brazil (between municipalities within the same state, between municipalities in different states); (2) determining sources and sinks of malaria infections occuring in Brazil between 2004 and 2022 and their temporal stability; and (3) analyzing spatiotemporal clusters of sources, sinks, and high movement regions.

## Methods

### Data

We leveraged two de-identified, government collected datasets from Brazil: the Malaria Epidemiological Surveillance Information System (Sivep-Malaria) and the Notifiable Disease Information System (SINAN) for the years 2004 to 2022. De-identified data were obtained through official communication channels with colleagues at the NMCP.

We define a malaria case as any positive malaria test result collected via official government testing facilities, excluding relapses. While Sivep-Malaria has all cases reported in Brazilian Amazon, SINAN has information on extra-Amazon cases. Data for the year 2003 are available only in Sivep-Malaria, and thus that year was excluded from the analysis. Most reported cases are confirmed through either a rapid diagnostic test (RDT) or blood smear microscopy, while a small number are confirmed via polymerase chain reaction. Here, we limit our analysis to cases where the infection occurs within Brazil.

Both SINAN and Sivep-Malaria contain variables that allow identifying imported cases. These include the country/state/municipality of notification, country/state/municipality of infection, and country/state/municipality of residence (Brazil has 26 states and one federal district, and is divided into 5,570 municipalities, 772 of those in the Legal Brazilian Amazon [a designation for socioeconomic development purposes of the land area of Brazil that sits with the Amazon Rainforest ecosystem]). The NMCP considers all municipalities in the states of Rondônia, Acre, Amazonas, Roraima, Pará, Amapá, Maranhão, Tocantins, and Mato Grosso to comprise the Brazilian Amazon, and we use this definition in our analysis. Following criteria used by the NMCP and further specified through the framework presented by Arisco et al. [11], we defined movement types based on infection and notification areas, not considering location of residence [11, 35]. We removed any case records in which a municipality of infection or notification were not listed (n = 123,345; 2.2% of all records). A locally acquired case is one in which the notification municipality is the same as the infection municipality, while an imported case is one in which the notification municipality is different from the infection municipality and is quantified based on the notification municipality. An exported case is one in which the notification and infection municipalities are different, but is quantified by the infection municipality. We further detailed movements as between- or within-state. Those definitions were applied at two different spatial scales: states (N = 27) and municipalities (N = 5,570). We used time series decomposition to assess whether the seasonal component of locally acquired and imported malaria cases had distinct patterns. To do this, we remove the trend and random noise from the time series of cases for each state and graphed the seasonal multiplicative component of the time series.

Additional variables used in this analysis (only available in Sivep-Malaria) include locality type of infection (urban, *garimpo* [or artisanal mining], settlement, indigenous, rural), date of symptom onset, date of infection, age, sex, parasite species, malaria detection format (active [systematic screening in high-risk areas] or passive [careseeking individuals entering clinics for treatment]), and type of occupation. In Sivep-Malaria and SINAN, some cases are designated as treatment verification slides, i.e. a check for relapses. These cases were removed from the dataset so that cases included were those who had not yet received treatment (n = 1,079,551). We calculated the Annual Parasite Index (API) as the number of confirmed malaria cases in the municipality of infection divided by the population of the municipality multiplied by 1,000. We followed the NMCP criteria to categorize the API [36]: no malaria, API = 0; very low, 0>API<1; low, 1≥API<10; medium, 10≥API<50; and high, API≥50.

Both untreated and asymptomatic cases may not be captured by the malaria surveillance systems in Brazil for several, including limited health-care access or lack of symptoms requiring treatment. Certain traits of the Brazilian health system serve to reduce the likelihood of missed malaria cases, including free provision of health care, government-exclusive distribution of malaria treatment through malaria treatment facilities, and the implementation of active case detection in isolated regions with limited healthcare access and higher rates of transmission. However, health care in some isolated areas (e.g., indigenous protected areas) remains limited [35, 37].

### Network analysis and typology of sources and sinks of malaria

Different methods have been used to collect data on the movement of malaria-infected individuals. Most commonly, movement has been measured using travel recall data from surveys and, more recently, mobile-phone call record data—both of which are then combined with malaria prevalence data at some spatial scale [5, 38]. Malaria infection rates are then inferred using mathematical models, which are coupled with human movement data to provide mobility patterns of infected individuals. Despite limitations, these methods have proven useful, especially in countries that do not systematically collect data and/or lack a health information system [39].

In the case of Brazil, movement of malaria-infected individuals can be measured more directly through Sivep-Malaria and SINAN. We leveraged daily, individual-level data and aggregated it to the monthly, municipality level which we then used in a network analysis [40] to create mobility networks of malaria-infected individuals. Organizing data as a directional network allows for direct measurement of flows of malaria cases and connectivity of geographical locations and has proven effective in informing the stratification of malaria control efforts [41]. These measurements are useful to characterize mobility in the context of malaria transmission and to generate mobility variables that can be incorporated into explanatory and multivariable modeling frameworks [42].

In each network, municipalities were designated as nodes, and a directional tie was defined as an individual becoming infected in one node and traveling to another node for notification. The strength of ties was denoted as the number of individuals traveling along the defined route in each period. We assessed both the degree of each node (the number of locations each municipality either sends cases to, defined as out-degree, or receives cases from, defined as in-degree), and the strength of each node (the number of cases each municipality sends—out-strength, or receives—in-strength) at monthly intervals between 2004 and 2022. Mobility networks of infected individuals were created for within- and between-state movements. All analyses were conducted in R v4.0.0 using the “network” package [43].

Nodes in the network were designated as sources, sinks, or both. Sources were municipalities that distributed cases to other municipalities, while sinks were municipalities that received cases from other municipalities. We classified nodes by both their mean in- and out-degree and mean in- and out-strength over the study period. Based on the highly right-skewed distribution, we apply an extreme percentile cutoff to designate high (≥99^th^ percentile) and low (<99^th^ percentile) intensity: (A) high strength, high degree; (B) low strength, high degree; (C) high strength, low degree; (D) low strength, low degree. These categories were detailed for between- and within-state movements. Sources in Type A were designated super-spreaders, while sinks in that category were designated super-receivers. The stability of sources and sinks is assessed across time by calculating the coefficient of variation (CV) of the cases leaving/entering the municipality each month, such that municipalities with higher CVs have more stable monthly cases exported or imported.

Lastly, we captured total movement in and out of an area in each period by extending the demographic concept of the gross migration rate to malaria cases in each municipality, in other words, we calculated the gross case migration rate (GCMR) = (imported malaria cases + exported malaria cases)/(Mid-year population)*1,000.

### Summary of mobile case traits

We stratify cases by demographic characteristics of individuals with locally acquired infections, those moving between-states, and those moving within-states using all available Sivep-Malaria data from 2004–2022 (n = 4,563,654 cases) (Table C in S1 Text). This analysis was restricted to states in the Amazon region, which concentrated more than 99% of cases in Brazil. Demographic characteristics include age category (<5, 5–15, 16–24, 25–40, 41–64, ≥65 years), sex (female, male), occupation (binary indicator for artisanal mining (garimpo), forestry, agriculture, domestic, or hunter/fisher), parasite species (*Plasmodium falciparum*, *Plasmodium vivax*, other/mixed infection), detection format (active, passive), locality type of infection (binary indicator for indigenous, rural, artisanal mining (garimpo), settlement), month of the year, and time to care (difference between symptom onset and diagnosis based on Pan-American Health Organization classifications; <24 hours (reference), 24–48 hours, >48 hours).

### Space-time clusters

Again we aggregated daily, individual data to the monthly, municipality level. Retrospective space-time clusters of sources, sinks, and high movement areas were determined by using the Kulldorf spatial scan statistic [44] based on a Poisson model and further detailed by within- and between-state movements. We stratified this analysis by year to minimize year-to-year distributional variation and to assess finer temporal resolution clusters within each year. A circular spatial scan window, a maximum spatial cluster size of 5% of the population, and a temporal cluster range allowance of 2–6 months were used. For the clustering of GCMR values, a normal model was used to accurately reflect the continuous distribution of values. The statistical significance of clusters was tested via likelihood ratio tests, with P-values based on 9,999 Monte Carlo replications (999 for normal models). To correct for multiple testing, we implement a Bonferroni correction, such that the α value for Poisson models was equal to 0.05/(19*4) = 0.0006 and the α value for normal models was set to the minimum P-value possible via monte carlo repetitions, 0.001. Any cluster with P≤ α was considered statistically significant. Cluster analyses were run on SaTScan v9.6 [44].

We performed all demographic analyses, data cleaning, and processing in R v.4.0.0 (R core team, 2020). We created data visualizations in R, ArcMap v.10.8 (ESRI; Redlands, CA), and Microsoft PowerPoint.

## Results

### Malaria transmission from 2004 to 2022

**Fig 1:**
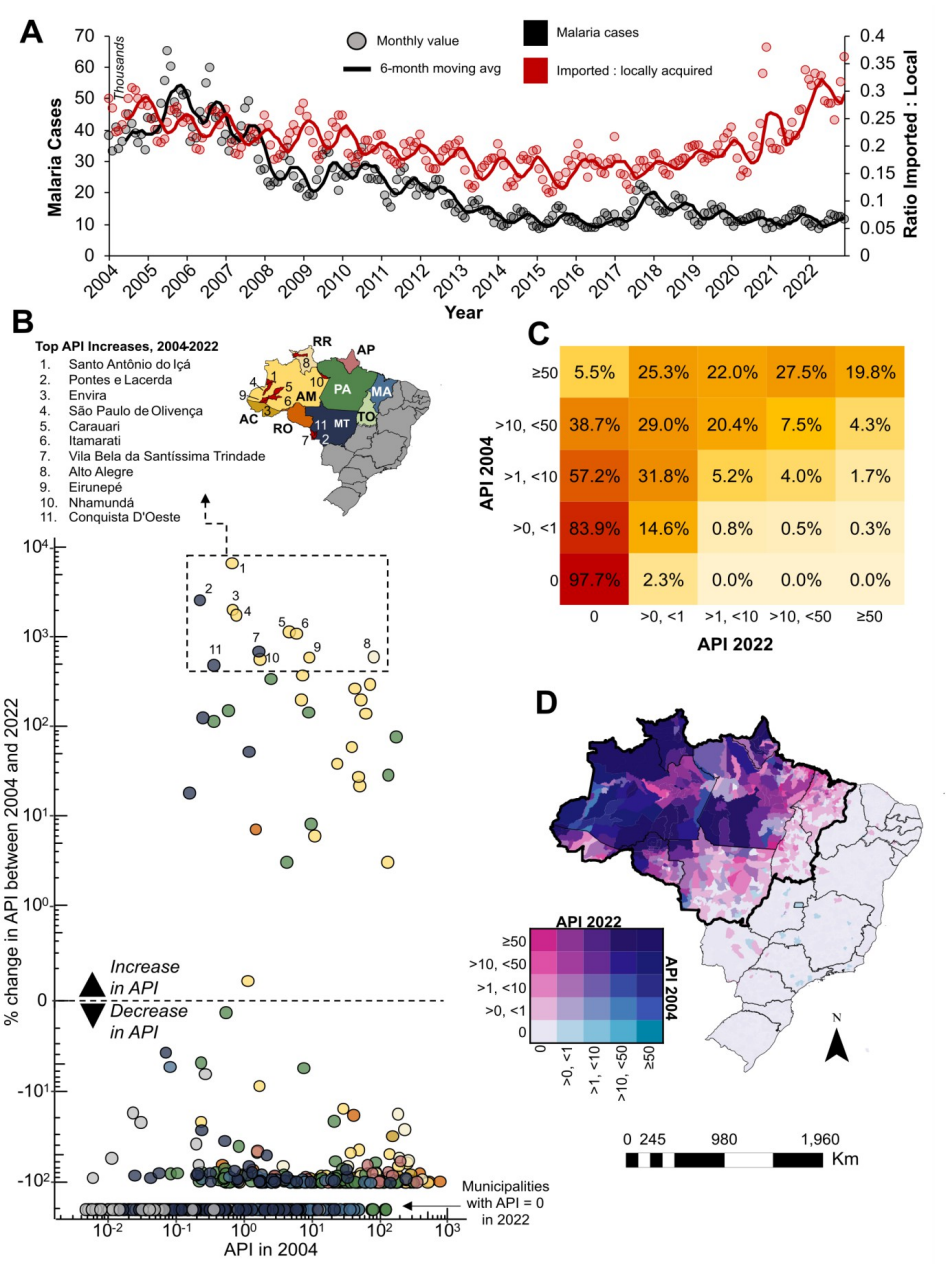
Trends in annual parasite index (API) and imported malaria cases in Brazil from 2004 to 2022. **(A)** Monthly and 6-month moving average of total malaria cases (black) and ratio of imported to locally acquired malaria cases (red). **(B)** Percent change in API from 2004 to 2022 compared to 2004 values for all municipalities, benchmarked against average change. The top 10 municipalities with the largest changes are shown on the map. Point colors correspond to their respective states. State acronyms: AC = Acre, AP = Amapá, AM = Amazonas, PA = Pará, RO = Rondônia, RR = Roraima, TO = Tocantins, MA = Maranhão, and MT = Mato Grosso. **(C)** Proportion of municipalities in categories of API in 2022 compared to 2004. API values are categorized using NMCP criteria. **(D)** Bivariate map of API in 2004 and 2022, by municipality. States outlined in darker black designate those in the Brazilian Amazon. Shapefile source: https://www.ibge.gov.br/en/geosciences/territorial-organization/territorial-meshes/18890-municipal-mesh.html?edicao=33161&t=o-que-e.

A total of 4,978,548 malaria cases were reported to originate in Brazil between January 2004 and December 2022. After an annual peak of 599,229 cases in 2005, a declining trend in cases was observed, except for small rebounds in 2010 and 2021, and a larger one in 2017–2018 (Fig 1A). On average, the API declined by almost 53.3% between 2004 and 2022, with seven of the ten municipalities with the largest increases in that period located in Amazonas state (Fig 1B). Also, 19.8% of the municipalities that had API≥50 in 2004 were in that category in 2022 (Fig 1C). The majority of municipalities with low API in both years are located at the edge of and outside the Amazon region (Fig 1D), and this was consistent across the study period (Fig A in S1 Text).

### Patterns of imported malaria

Annually, on average, 17.5% (min: 12.3% in 2015, max: 23.1% in 2022) of malaria cases reported by municipalities were imported. Extra-Amazonian states had, on average, 79.3% of all cases imported annually, while this number was 17.4% for Amazonian states (with Tocantins being the highest at 79.4%). Importation mainly occurred within the same state (ranging from 68.1% in 2021 to 84.9% in 2010) (Table A in S1 Text). Seasonal patterns were largely similar, except for imported cases between states. Maranhão, with lower API and located at the edge of the Amazon region, had the most distinct seasonal pattern between locally acquired and imported cases (Fig B in S1 Text).

**Fig 2:**
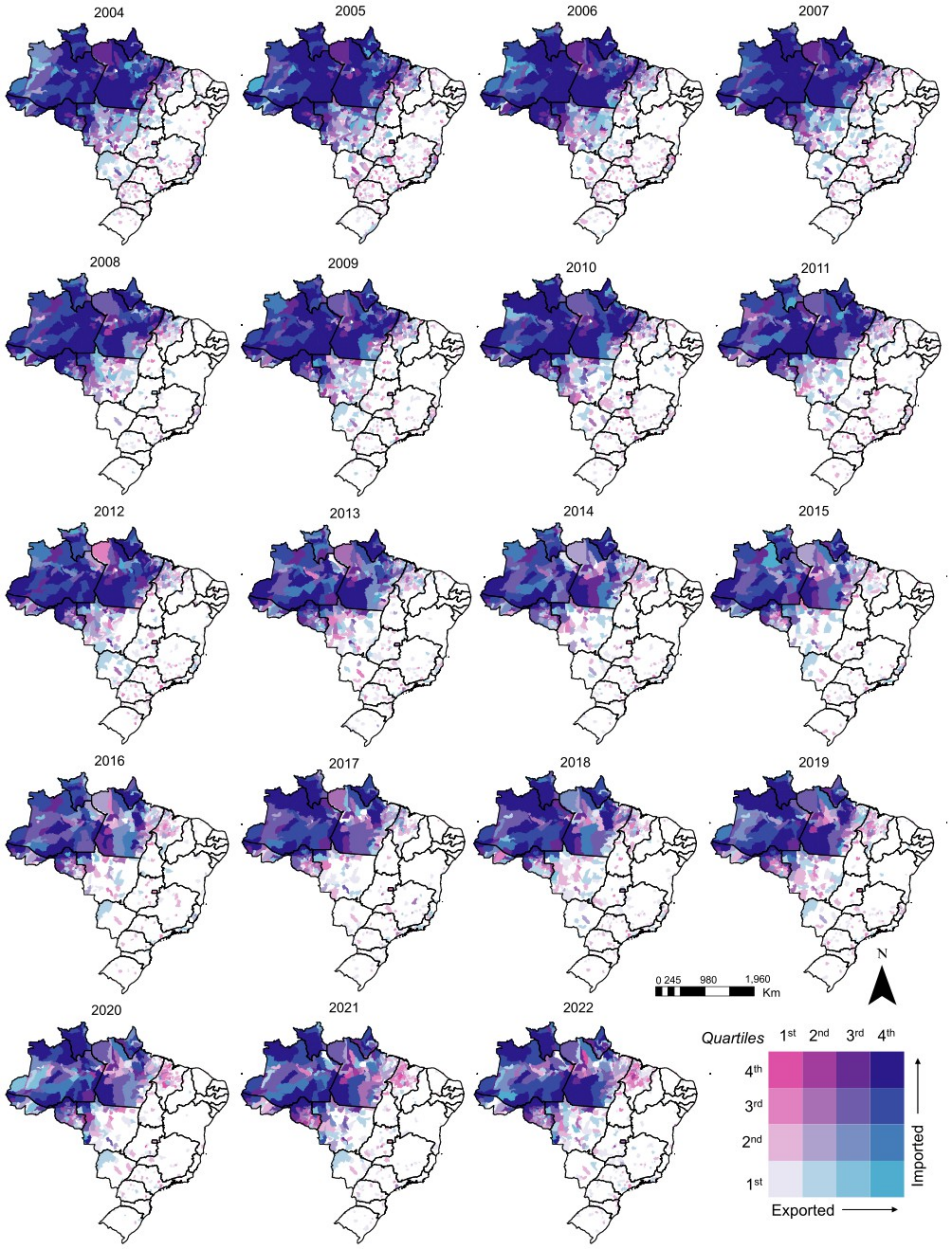
Bivariate maps of the annual number of malaria cases imported to and exported from each municipality in Brazil, 2004 to 2022. Bivariate distribution based on quartiles. Imported quartiles: Q1≤1.0, 1.0<Q2≤3.0, 3.0<Q3≤18.0, Q4>18; Exported quartiles: Q1 = 0, 0<Q2≤1, 1<Q3≤12, Q4>12. Shapefile source: https://www.ibge.gov.br/en/geosciences/territorial-organization/territorial-meshes/18890-municipal-mesh.html?edicao=33161&t=o-que-e.

**Fig 3:**
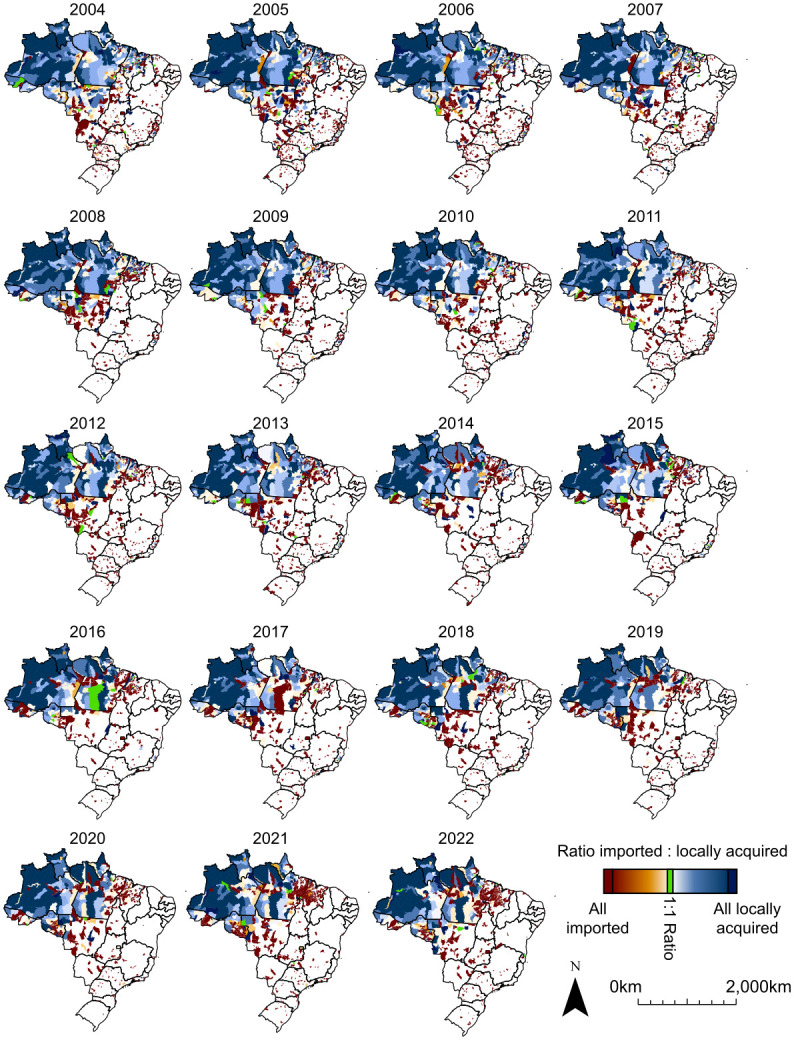
Annual ratio of imported to locally acquired cases in each municipality in Brazil, 2004 to 2022. Shapefile source: https://www.ibge.gov.br/en/geosciences/territorial-organization/territorial-meshes/18890-municipal-mesh.html?edicao=33161&t=o-que-e.

The spatial pattern of imported malaria reveals that some municipalities in the Amazon receive a large number of imported cases but are also the source of cases exported to other locations. In contrast, municipalities at the edge of the Amazon region (mainly those in the states of Maranhão, Tocantins, and Mato Grosso), and those outside of the Amazon, receive imported cases but rarely are the source of infection for cases notified in other municipalities (Fig 2). To further investigate that pattern, we considered a ratio relating imported and locally acquired cases (RILA). The ratio shows that there is a core area in the Amazon where, despite the high volume of malaria cases imported and exported to other areas, the bulk of reported cases are locally acquired, while in other municipalities malaria cases are mainly imported (Fig 3). During the study period, municipalities with low RILA typically reported the largest API (Fig 3 and Fig A in S1 Text). In 2022, for example, the average API in municipalities that had more locally acquired cases than imported ones was 25.9 cases per 1,000 population, and those that had more imported cases was 18.7 cases per 1,000 population.

### Municipal network of importation

Considering within-state importation, the most common route over the entire study period was from the municipality of Alto Alegre to Boa Vista in Roraima (2.9% of all within-state monthly routes between 2004 and 2022). This route was also the most common importation route, and Alto Alegre consistently had a higher API than Boa Vista (2.4% of all importation routes over the study period). As for between-state routes, the most frequent was from Canutama, in Amazonas, to Porto Velho, in Rondônia (Distance between municipalities: 249.88km; 11.4% of all between-states monthly routes, and 2.1% of all importation routes between 2004 and 2022). While the top ten within-state routes in frequency accounted for 20.2% of all within-state monthly routes over the study period, the top ten between-states routes represented 44.1% of all between-states routes (Table B in S1 Text).

Of the 4,238,244 malaria cases in Sivep-Malaria from 2004–2022, 137,098 were between-state importation and 607,870 were within-state importation, compared to 3,493,276 locally acquired cases. 62.0% of individuals were male, the most commonly represented age group was individuals aged 5–15 years (26.2%), and the most common occupation was being an agricultural worker (25.2%). *P*. *vivax* was the parasite of infection in 81.9% of cases, while this percentage was slightly lower among mobile cases (79.5%). Males were far more commonly infected with malaria than females: 72.3% of between-state cases and 67.9% of within-state cases compared to 60.6% of locally acquired cases. Among locally acquired cases, the age group with the highest number of infections was 5–15, while in between-state and within-state the 25–40 age group was most highly represented. Being infected with either *P*. *falciparum* was more common among between-state movements (17.1%) as compared to within-state (15.4%) and locally acquired cases (13.7%). Between-state movements more commonly listed their occupation as garimpo (17.1%) compared to within-state (11.2%) and locally acquired cases (5.8%). Finally, being detected through passive detection was more common among imported cases than locally acquired cases, and a higher percentage of imported cases took >48 hours from symptom onset to time to care (Table C in S1 Text).

### Municipal sources and sinks of malaria

**Fig 4:**
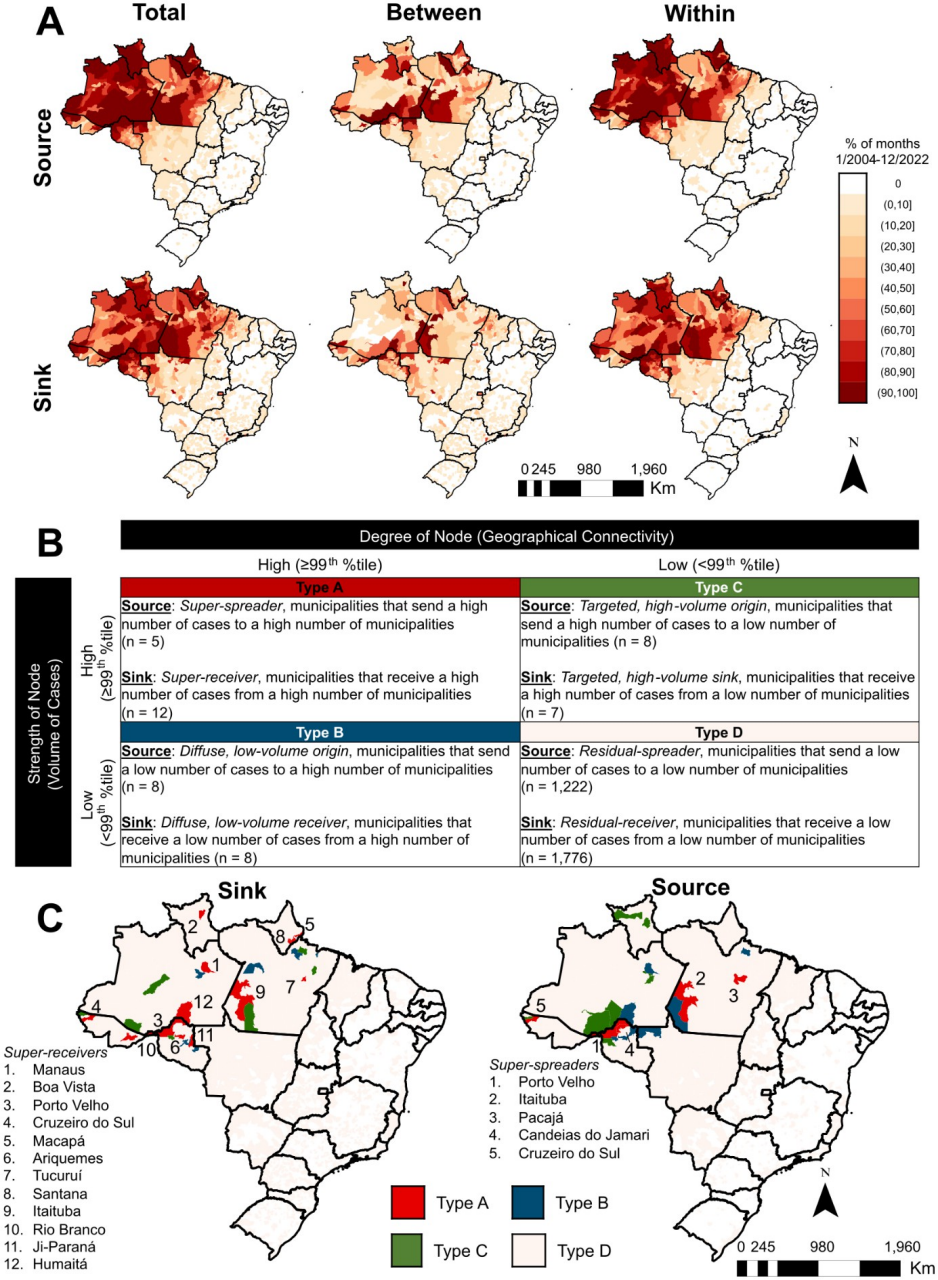
Typology of malaria source and sink municipalities. **(A)** Percentage of months between 2004 and 2022 that each municipality was a malaria source or sink, detailed by all movements, between-state movements, and within-state movements. **(B)** Proposed typology of municipalities considering the malaria case importation pattern. **(C)** Map of typology of municipalities by source and sink. Super-spreaders and super-receivers are labeled. Shapefile source: https://www.ibge.gov.br/en/geosciences/territorial-organization/territorial-meshes/18890-municipal-mesh.html?edicao=33161&t=o-que-e.

Over the study period, monthly importation of malaria cases was more frequent among municipalities in the same state, while importation between states was more common among municipalities sharing a border (Fig 4A).

Municipality degree and strength varied spatiotemporally, though municipalities with the highest degree and strength on average across the study period were in the state of Amazonas, Roraima, and Rondônia. We assessed the stability of sources and sinks across time by calculating the coefficient of variation (CV) of malaria cases leaving (source) or entering (sink) municipalities each month over the study period. Sources of malaria cases were more stable than sink municipalities between 2004 and 2022 (Fig D in S1 Text). Five municipalities across the Amazon region were classified as super-speaders and twelve were classified as super-recievers (Fig 4B and 4C, Table D in S1 Text). Delineating by within- and between-state movements, we found that over the study period, the top three most consistent (highest CV) within-state super-speader municipalities were Itaituba (Pará), Candeias do Jamari (Rondônia), and Porto Velho (Rondônia), while the most consistent between-state super-spreader municipalities were Itaituba (Pará), Porto Velho (Rondônia), and Humaitá (Amazonas). Concerning super-receivers, the most temporally consistent municipalities for within-state movements were Macapá (Amapá), Porto Velho (Rondônia), and Boa Vista while the most consistent between-state super-receiver municipalities were Manaus (Amazonas), Boa Vista (Roraima), and Itaituba (Pará). The state that had the highest average out-degree for both between- and within-state movements was Roraima. Between-state importation of malaria was overwhelmingly represented by routes between Amazonas, Rondônia, and Acre (44.5% of all between movements), while within-state importation was more evenly distributed across malaria-endemic states in Brazil. Porto Velho (Rondônia) was commonly both a super-spreader and super-receiver.

### Intense case mobility municipalities

Since many municipalities receive and export malaria cases at high numbers (Fig 2), and some are both super-spreaders and super-receivers (Fig 4C), we calculated a gross case migration rate (GCMR) that combines cases imported and exported and thus quantifies the volume of malaria cases moving in and out of a municipality each month. We observed a positive correlation between GCMR and API (Fig D in S1 Text, Table E in S1 Text). The 20 municipalities with the highest GCMR between 2004 and 2022 had an average API of 159.1 cases per 1,000 people, and are mainly in the states of Acre, Amazonas, Amapá, Pará, Rondônia, and Roraima. The bottom 20 municipalities had an average API of 0.00002 cases per 1,000 people, and are located in extra-Amazonian states (Table F in S1 Text).

### Spatiotemporal clustering

**Fig 5:**
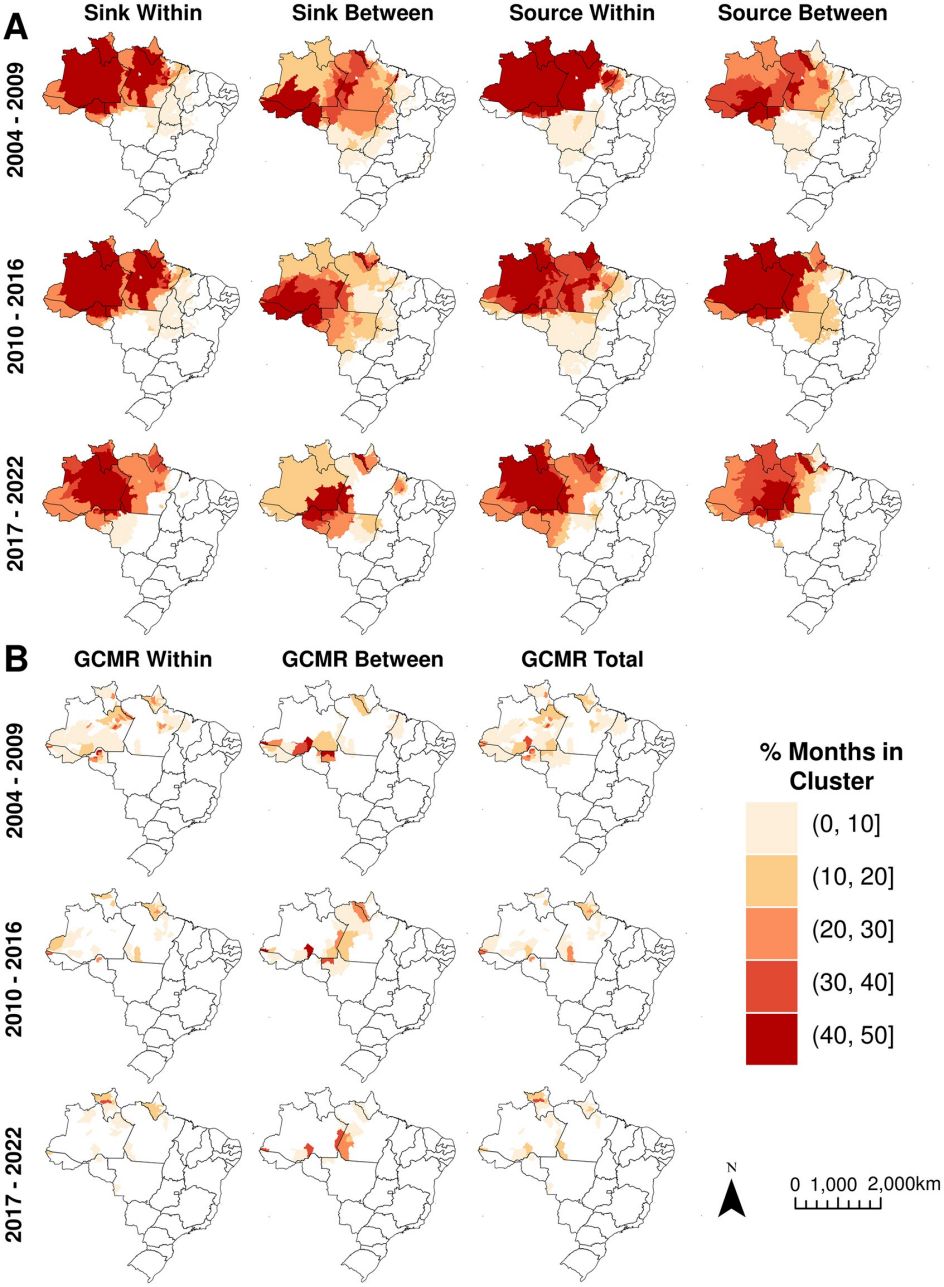
Spatiotemporal clustering by period and movement type. The color scale represents the percentage of months each municipality was in a statistically significant cluster. **(A)** Clustering of monthly sink and source values within and between states. **(B)** Clustering of monthly GCMR within states, between states, and total movement. Shapefile source: https://www.ibge.gov.br/en/geosciences/territorial-organization/territorial-meshes/18890-municipal-mesh.html?edicao=33161&t=o-que-e.

To assess whether municipalities identified as sources, sinks, and intense mobility of infected individuals clustered in space and time we used the spatiotemporal scan statistic stratified by each year in the dataset [44]. In total, 151 statistically significant (P≤0.0006) clusters of source municipalities (65 between-state, 86 within-state) and 171 clusters of sink municipalities (97 between-state, 74 within-state) were detected. Source clusters, on average, persisted for 4.77 months, while sink clusters lasted an average of 4.73 months. Broadly, a core group of municipalities persisted as clusters of sources of malaria importation, especially until 2016 (Fig 5A and Table G in S1 Text). Regarding intense case mobility municipalities, there were 208 within-state GCMR, 124 between-state GCMR, and 236 total GCMR statistically significant clusters (P≤0.001) (Fig 5B and Table H in S1 Text), with a shorter duration than sources and sinks, an average of 2.99 months (SD: 0.84) for within-state GCMR, 4.14 months (SD: 0.77) for between-state, and 2.79 months (SD: 0.73) for all movements.

On average, municipalities that tested significant for clusters had higher API compared to the mean API of all municipalities (Fig E in S1 Text). Similarly, the API of municipalities was negatively associated with the RILA and this relationship varied by state (Fig E in S1 Text).

## Discussion

Our results quantify the movement and seasonality of malaria cases between- and within-states in Brazil, demonstrate key demographic risk factors for imported malaria cases in the Amazon region, and highlight geographical regions where intense case mobility has been consistently high. We show that most importation occurred within states and that a small number of within-state routes accounted for nearly one-quarter of the movement of malaria-infected individuals. We also show that a core group of municipalities in the center of the Brazilian Amazon persisted as clusters of sources of malaria, while the eastern edge of the Amazon and extra-Amazonian states receive imported cases but rarely are the source. The metrics and analytical framework we developed for malaria case mobility, drawn directly from routinely collected data, demonstrate the importance of incorporating importation in malaria elimination plans.

The spatiotemporal patterns of malaria case mobility revealed in this study augment the capabilities of the Brazilian NMCP to employ intervention stratification in line with the WHO Global Technical Strategy for Malaria [45]. Specifically, we identified five important characteristics of malaria importation in Brazil that could improve actions by the NMCP. First, malaria case mobility is seasonal, and certain population groups dominate the importation of cases. Second, municipalities characterized by high case mobility are temporally consistent. Third, importation contributes to maintaining residual malaria transmission in municipalities with low API. Fourth, few inter-municipality routes account for a large portion of all case mobility. Fifth, most municipalities in the Brazilian Amazon are characterized by low-volume movements to few municipalities. Those five characteristics provide evidence for targeted and intensified interventions during certain times of the year, in certain areas, directed to specific population groups. In areas in the southern and eastern edges of the Amazon (in the states of Mato Grosso, Maranhão, and Tocantins), those targeted interventions will be critical to achieving sub-national elimination and ultimately place Brazil on track to achieve its malaria elimination goal. Moreover, transmission is also concentrated in a few municipalities; in 2022, 30 municipalities accounted for 80% of malaria cases, and 80% of *P*. *falciparum* cases were concentrated in 16 municipalities.

Seasonality of mobility of malaria infected individuals is often driven by economic opportunity (i.e., mining, deforestation, agriculture) [27, 46–48]. These activities often begin with clearcutting the forest, which creates suitable habitats for *Nyssorhynchus (Anopheles) darlingi*, following the early patterns of frontier malaria [19, 49]. Not only does this ecological change increase malaria risk in individuals directly involved in those activities, but it also alters the exposure among residents of nearby areas directly affected [47]. In addition, mobile cases traveling between municipalities with high API where *garimpo* is happening [50], are far more likely to be detected via passive than active surveillance. This presents an opportunity for the NMCP to intensify active surveillance along commonly traveled routes, as well as in areas with new environmental disturbance as detected by alerts from the Brazilian National Institute for Space Research’s Deforestation Detection in Real Time (INPE-DETER) program [51]. Cross-sectoral collaboration between the NMCP, Brazil’s Ministry of Environment and Climate Change, and the Department of Environmental Health and Occupational Health Surveillance may facilitate these novel forms of surveillance.

Our results show that the foci of malaria case mobility have changed over time. Between 2004 and 2008, the mobility of cases was most common in the state of Rondônia but shifted to Pará starting in 2009. Though our analyses do not explicitly uncover why foci shift over time, historically economic forces such as land-grabbing, settlement, extended urbanization, and economic opportunity including *garimpo* may drive intense mobility [19, 22]. Over the study period, between-state mobility of infected individuals increased relative to within-state mobility but started to decline in 2020 with the onset of the Covid-19 pandemic. Between-state mobility of infected individuals in the Brazilian Amazon is particularly complex, as some individuals may travel shorter distances for care between states rather than within their state of infection (especially in the case of municipalities that are at the state border).

Regarding sources and sinks of malaria, we show that some municipalities are uniquely classified as a source or a sink, while other municipalities are both. Porto Velho, the capital of Rondônia, is a municipality that extends throughout the northern portion of the state and has a wide variety of ecological contexts, such as urban, forest fringe, agricultural settlements, and newly deforested areas. Porto Velho was the largest, most consistent top-origin and top-receiver municipality [30, 52]. It receives an extremely high volume of malaria cases from many municipalities (primarily in Amazonas state) while sending a high volume of cases to many municipalities in Acre and Amazonas. Infrastructure projects and tourism have been shown to drive mobility in and out of the municipality at high rates [30]. In riverine localities of Porto Velho, epidemics of malaria have broken out in migrants seeking economic opportunity, but with little prior malaria exposure [52]. Migration brought about by this spike in economic opportunity was facilitated by travel networks such as BR-364, a major roadway through the Amazon, which connects rural areas to urban centers [53, 54].

This study has many strengths. We use the longest time series with the finest spatial scale to characterize the mobility of malaria cases in the Brazilian Amazon. Our results not only advance current knowledge but also inform the need to target interventions in time, space, and population groups to curb the effects of mobility, contributing to current elimination goals. In addition, the metrics and typology presented in this study can be automated and serve as a surveillance tool for the NMCP.

This study has some limitations. First, data on the most likely location of infection are based on travel recall and the onset of symptoms, which may be subject to recall bias. However, inaccuracies in individual recall are expected to be minimal since most cases receive treatment within a very short time reference. Given the large amount of data analyzed here, we maintain confidence in case mobility estimates representing true dynamics. Second, asymptomatic malaria infections may not be fully captured by the NMCP surveillance despite active surveillance measures, and this is true for any malaria surveillance system. Third, new *P*. *vivax* infections may not be fully distinguishable from relapses given the dormant behavior of the parasite. In addition, although *P*. *vivax* cases are treated with chloroquine (for 3 days) and primaquine (7 days) [55], some patients may not complete the treatment regimen as recommended [56]. Fourth, SatScan may be subject to multiple testing. However, we incorporate conservative statistical significance cutoffs to handle the potential of type I errors.

In summary, in-country importation of malaria is a challenge to malaria elimination in Brazil [12]. Vast travel networks characterize the Amazon region, and the ability to understand and monitor the temporal and spatial dynamics of malaria case mobility is vital for effective microstratification to support elimination targets [23, 57]. Here, we provide evidence, metrics, and methodology to support NMCP’s actions toward malaria elimination.

## Supporting information

S1 Text Table A. Total and imported malaria cases (only between/within-states in Brazil), detailed by within- and between-state movements for all of Brazil, 2004–2022. Table B. Top 10 most common malaria importation routes in Brazilian municipalities considering the years 2004 to 2022. State acronyms: AC = Acre, AM = Amazonas, PA = Pará, RO = Rondônia, RR = Roraima, and MT = Mato Grosso. Table C. Summary groupings of Sivep-Malaria. The analysis includes only states in the Brazilian Amazon (Acre, Rondônia, Amazonas, Roraima, Pará, Amapá, Tocantins, Maranhão, and Mato Grosso) for the years 2004 to 2022. Table D. Top source and sink municipalities of malaria importation by node type. Types were defined in Methods and Fig 4B. Type D is the most common and the table includes only the top 15 municipalities. Table E. Annual Pearson correlation between the Gross malaria Case Migration Rate (GCMR) and the Annual Parasite Index (API) for all municipalities in Brazil. LCI = Lower bound of 95% confidence interval. UCI = Upper bound of 95% confidence interval. Table F. Top and bottom 20 municipalities for mean Gross malaria Case Migration Rate (GCMR) and mean Annual Parasite Index (API), Brazil 2004–2022. Table G. Spatio-temporal analysis of within- and between-state sources and sinks of malaria-infected individuals. Clustering was assessed with the Scan Statistic and only clusters with P<0.0006 were included in the table (Methods). Table H. Clustering analysis of Gross Case Migration Rate (GCMR) of infected individuals, using the Scan Statistics. Only clusters with P≤0.001 were included (Methods). Fig A. Annual Parasite Index, Brazil 2004–2022. Calculated as the number of confirmed malaria cases in the municipality of infection divided by the population of the municipality multiplied by 1,000. Shapefile source: https://www.ibge.gov.br/en/geosciences/territorial-organization/territorial-meshes/18890-municipal-mesh.html?edicao=33161&t=o-que-e. Fig B. Seasonal multiplicative pattern of locally acquired and imported malaria cases by month, Brazil 2004–2022. The seasonal multiplicative component was obtained by time series decomposition, removing trend and random noise (Methods). State acronyms: AC = Acre, AP = Amapá, AM = Amazonas, PA = Pará, RO = Rondônia, RR = Roraima, TO = Tocantins, MA = Maranhão, and MT = Mato Grosso. Fig C. Boxplot of the coefficient of variation for each mobility flow. Calculation based on monthly municipal data for the years 2004 to 2022. Fig D. Malaria by period, Brazil 2004–2022. (Top) Boxplots of the Annual Parasite Index considering municipalities that were part of significant spatiotemporal clusters, by cluster type and period. (Bottom) Regression lines and R2 between the ratio relating imported and locally acquired cases (RILA) and API, by state and period. The state of Tocantins has a large confidence interval in 2017–2022 because it reported very few malaria cases during that period. Fig E. Correlation between the Gross malaria Case Migration Rate (GCMR) and the Annual Parasite Index (API). All Brazilian municipalities and years 2004–2022.
(DOCX)
