# Supplementary material for: Spatiotemporal analysis of within-country imported malaria in Brazilian municipalities, 2004–2022
Source: PLOS Glob Public Health. 2024 Jul 15;4(7):e0003452. doi: 10.1371/journal.pgph.0003452 (PMC11249269; doi:10.1371/journal.pgph.0003452)

**Online Supplement**

**Sources and sinks of malaria in Brazilian municipalities, 2004-2022**

Nicholas J. Arisco, PhD^1^; Cassio Peterka, MS^2^; Marcia C Castro, PhD^1*^

^1^ Department of Global Health and Population, Harvard TH Chan School of Public Health, Boston, MA 02115, USA.

^2^ Secretaria de Vigilância em Saúde e Ambiente, Ministério da Saúde, Brasília, DF, 70723-040, Brazil

*Correspondence to: [mcastro@hsph.harvard.edu](mailto:mcastro@hsph.harvard.edu)

**Contents**

[Table A in S1 Text. Total and imported malaria cases (only between/within-states in Brazil), detailed by within- and between-state movements for all of Brazil, 2004-2022. 3](#_Toc141362634)

[Table B in S1 Text. Top 10 most common malaria importation routes in Brazilian municipalities considering the years 2004 to 2022. 4](#_Toc141362635)

[Table C in S1 Text. Summary groupings of Sivep-Malaria. 5](#_Toc141362636)

[Table D in S1 Text. Top source and sink municipalities of malaria importation by node type. 6](#_Toc141362637)

[Table E in S1 Text. Annual Pearson correlation between the Gross malaria Case Migration Rate (GCMR) and the Annual Parasite Index (API) for all municipalities in Brazil. 7](#_Toc141362638)

[Table F in S1 Text. Top and bottom 20 municipalities for mean Gross malaria Case Migration Rate (GCMR) and mean Annual Parasite Index (API), Brazil 2004-2022. 8](#_Toc141362639)

[Table G in S1 Text. Spatio-temporal analysis of within- and between-state sources and sinks of malaria-infected individuals. 9](#_Toc141362640)

[Table H in S1 Text. Clustering analysis of Gross Case Migration Rate (GCMR) of infected individuals, using the Scan Statistics. 10](#_Toc141362641)

[Figure A in S1 Text. Annual Parasite Index, Brazil 2004-2022. 11](#_Toc141362642)

[Figure B in S1 Text. Seasonal pattern of locally acquired and imported malaria cases by month, Brazil 2004-2022. 12](#_Toc141362643)

[Figure C in S1 Text. Boxplot of the coefficient of variation for each mobility flow. 13](#_Toc141362644)

[Figure D in S1 Text. Malaria by period, Brazil 2004-2022.. 14](#_Toc141362645)

[Figure E in S1 Text. Correlation between the Gross malaria Case Migration Rate (GCMR) and the Annual Parasite Index (API). 15](#_Toc141362646)

# Table A in S1 Text. Total and imported malaria cases (only between/within-states in Brazil), detailed by within- and between-state movements for all of Brazil, 2004-2022.

| **Year** | **Total** | | | **Within-State** | | | **Between-State** | | |
| --- | --- | --- | --- | --- | --- | --- | --- | --- | --- |
|  | **Cases** | **Imported Cases** | **% of total** | **Cases** | **% of imported** | **% of total** | **Cases** | **% of imported** | **% of total** |
| 2004 | 466,808 | 97,634 | 20.92 | 82,252 | 84.25 | 17.62 | 15,382 | 15.75 | 3.30 |
| 2005 | 608,190 | 117,801 | 19.37 | 100,039 | 84.92 | 16.45 | 17,762 | 15.08 | 2.92 |
| 2006 | 551,113 | 104,485 | 18.96 | 86,343 | 82.64 | 15.67 | 18,142 | 17.36 | 3.29 |
| 2007 | 459,562 | 80,299 | 17.47 | 66,124 | 82.35 | 14.39 | 14,175 | 17.65 | 3.08 |
| 2008 | 315,169 | 54,564 | 17.31 | 43,825 | 80.32 | 13.91 | 10,739 | 19.68 | 3.41 |
| 2009 | 309,027 | 54,040 | 17.49 | 45,147 | 83.54 | 14.61 | 8,893 | 16.46 | 2.88 |
| 2010 | 334,433 | 53,895 | 16.12 | 45,718 | 84.83 | 13.67 | 8,177 | 15.17 | 2.45 |
| 2011 | 294,890 | 46,488 | 15.76 | 39,034 | 83.97 | 13.24 | 7,454 | 16.03 | 2.53 |
| 2012 | 276,477 | 42,104 | 15.23 | 34,280 | 81.42 | 12.40 | 7,824 | 18.58 | 2.83 |
| 2013 | 205,512 | 27,325 | 13.30 | 21,338 | 78.09 | 10.38 | 5,987 | 21.91 | 2.91 |
| 2014 | 163,693 | 22,583 | 13.80 | 18,377 | 81.38 | 11.23 | 4,206 | 18.62 | 2.57 |
| 2015 | 165,528 | 19,987 | 12.07 | 16,324 | 81.67 | 9.86 | 3,663 | 18.33 | 2.21 |
| 2016 | 151,292 | 20,892 | 13.81 | 16,460 | 78.79 | 10.88 | 4,432 | 21.21 | 2.93 |
| 2017 | 237,820 | 31,079 | 13.07 | 24,422 | 78.58 | 10.27 | 6,657 | 21.42 | 2.80 |
| 2018 | 243,215 | 33,787 | 13.89 | 26,352 | 77.99 | 10.83 | 7,435 | 22.01 | 3.06 |
| 2019 | 189,824 | 30,437 | 16.03 | 22,355 | 73.45 | 11.78 | 8,082 | 26.55 | 4.26 |
| 2020 | 160,660 | 28,682 | 17.85 | 21,370 | 74.51 | 13.30 | 7,312 | 25.49 | 4.55 |
| 2021 | 163,208 | 32,262 | 19.77 | 21,720 | 67.32 | 13.31 | 10,542 | 32.68 | 6.46 |
| 2022 | 151,368 | 36,025 | 23.80 | 26,242 | 72.84 | 17.34 | 9,783 | 27.16 | 6.46 |

# Table B in S1 Text. Top 10 most common malaria importation routes in Brazilian municipalities considering the years 2004 to 2022. State acronyms: AC=Acre, AM=Amazonas, PA=Pará, RO=Rondônia, RR=Roraima, and MT=Mato Grosso.

| **Route** (Origein→Destination) | **State(s)** | **# of cases** | **% of total** |
| --- | --- | --- | --- |
| Routes of importation | | | |
| Alto Alegre→Boa Vista | RR | 24,647 | 2.64% |
| Canutama→Porto Velho | AM→RO | 19,038 | 2.04% |
| Cantá→Boa Vista | RR | 16,993 | 1.82% |
| Candeias do Jamari→Porto Velho | RO | 15,605 | 1.67% |
| Careiro→Manaus | AM | 14,481 | 1.55% |
| Porto Velho→Buritis | RO | 14,202 | 1.52% |
| Iranduba→Manaus | AM | 14,181 | 1.52% |
| Rodrigues Alves→Cruzeiro do Sul | AC | 13,723 | 1.47% |
| Guajará→Cruzeiro do Sul | AM→AC | 13,328 | 1.43% |
| Pacajá→Tucuruí | PA | 12,306 | 1.32% |
| Routes of within-state importation | | | |
| Alto Alegre→Boa Vista | RR | 24,647 | 3.25% |
| Cantá→Boa Vista | RR | 16,993 | 2.24% |
| Buritis→Porto Velho | RO | 15,605 | 2.06% |
| Careiro→Manaus | AM | 14,481 | 1.91% |
| Porto Velho→Buritis | RO | 14,202 | 1.87% |
| Iranduba→Manaus | AM | 14,181 | 1.87% |
| Rodrigues Alves→Cruzeiro do Sul | AC | 13,723 | 1.81% |
| Pacajá→Tucuruí | PA | 12,306 | 1.62% |
| Rio Preto da Eva→Manaus | AM | 12,179 | 1.61% |
| Moju→Tailândia | PA | 12,160 | 1.60% |
| Routes of between-state importation | | | |
| Canutama→Porto Velho | AM→RO | 19,038 | 10.78% |
| Guajará→Cruzeiro do Sul | AM→AC | 13,328 | 7.55% |
| Lábrea→Porto Velho | AM→RO | 8,195 | 4.64% |
| Almeirim→Laranjal do Jari | PA→AP | 6,916 | 3.92% |
| Maués→Itaituba | AM→PA | 6,199 | 3.51% |
| Itaituba→Maués | PA→AM | 5,369 | 3.04% |
| Maués→Jacareacanga | AM→PA | 4,814 | 2.73% |
| Colniza→Machadinho D'Oeste | MT→RO | 4,415 | 2.50% |
| Porto Velho→Rio Branco | RO→AC | 3,276 | 1.85% |
| Humaitá→Porto Velho | AM→RO | 3,108 | 1.76% |

# Table C in S1 Text. Summary groupings of Sivep-Malaria. The analysis includes only states in the Brazilian Amazon (Acre, Rondônia, Amazonas, Roraima, Pará, Amapá, Tocantins, Maranhão, and Mato Grosso) for the years 2004 to 2022.

| *2004-2022* | **Between-state** | | **Within-state** | | **Locally acquired** | |
| --- | --- | --- | --- | --- | --- | --- |
|  | **Total** | **Percent** | **Total** | **Percent** | **Total** | **Percent** |
| **Age group** | | | | | |  |
| <5 years | 6,842 | 5.0% | 43,215 | 7.1% | 372,057 | 10.7% |
| 5-15 years | 18,208 | 13.3% | 109,874 | 18.1% | 987,490 | 28.3% |
| 16-24 years | 29,845 | 21.8% | 132,425 | 21.8% | 694,740 | 19.9% |
| 25-40 years | 47,702 | 34.8% | 188,392 | 31.0% | 841,443 | 24.1% |
| 41-64 years | 31,817 | 23.2% | 120,619 | 19.8% | 523,012 | 15.0% |
| 65+ years | 2,684 | 2.0% | 13,345 | 2.2% | 74,534 | 2.1% |
| **Sex** | | | | | |  |
| Female | 37,976 | 27.7% | 195,078 | 32.1% | 1,376,406 | 39.4% |
| Male | 99,122 | 72.3% | 412,792 | 67.9% | 2,116,870 | 60.6% |
| **Occupation** | | | | | |  |
| Agriculture | 25,494 | 18.6% | 170,464 | 28.0% | 873,609 | 25.0% |
| Domestic | 11,111 | 8.1% | 50,031 | 8.2% | 383,090 | 11.0% |
| Forestry | 5,672 | 4.1% | 13,248 | 2.2% | 48,510 | 1.4% |
| Garimpo | 25,495 | 18.6% | 61,196 | 10.1% | 126,859 | 3.6% |
| Hunting/fishing | 2,373 | 1.7% | 14,064 | 2.3% | 91,164 | 2.6% |
| Other | 66,953 | 48.8% | 298,867 | 49.2% | 1,970,044 | 56.4% |
| **Special area of infection** | | | | | |  |
| Indigenous | 5,675 | 6.1% | 30,413 | 5.6% | 443,764 | 15.2% |
| Rural | 59,997 | 64.3% | 381,031 | 70.3% | 1,957,106 | 67.2% |
| Garimpo | 20,771 | 22.3% | 60,906 | 11.2% | 169,383 | 5.8% |
| Settlement | 6,883 | 7.4% | 69,964 | 12.9% | 340,074 | 11.7% |
| **Parasite species** | | | | | |  |
| *P. vivax* | 106,176 | 77.4% | 486,041 | 80.0% | 2,880,629 | 82.5% |
| *P. falciparum* | 23,494 | 17.1% | 93,742 | 15.4% | 479,904 | 13.7% |
| Mixed/other | 7,428 | 5.4% | 28,087 | 4.6% | 132,743 | 3.8% |
| **Testing format** | | | | | |  |
| Passive testing | 123,581 | 90.1% | 547,389 | 90.1% | 2,556,180 | 73.2% |
| Active testing | 13,517 | 9.9% | 60,481 | 9.9% | 937,096 | 26.8% |
| **Month of year** | | | | | |  |
| January | 12,430 | 9.1% | 55,004 | 9.0% | 284,180 | 8.1% |
| February | 10,070 | 7.3% | 44,790 | 7.4% | 245,068 | 7.0% |
| March | 9,619 | 7.0% | 42,518 | 7.0% | 264,072 | 7.6% |
| April | 8,523 | 6.2% | 38,772 | 6.4% | 254,482 | 7.3% |
| May | 9,768 | 7.1% | 44,511 | 7.3% | 295,437 | 8.5% |
| June | 10,736 | 7.8% | 49,583 | 8.2% | 313,043 | 9.0% |
| July | 12,398 | 9.0% | 62,736 | 10.3% | 357,233 | 10.2% |
| August | 13,396 | 9.8% | 63,658 | 10.5% | 346,001 | 9.9% |
| September | 12,163 | 8.9% | 56,060 | 9.2% | 311,697 | 8.9% |
| October | 12,907 | 9.4% | 52,921 | 8.7% | 292,818 | 8.4% |
| November | 12,909 | 9.4% | 50,479 | 8.3% | 286,424 | 8.2% |
| December | 12,179 | 8.9% | 46,838 | 7.7% | 242,821 | 7.0% |
| **Time to care** | | | | | |  |
| <24hrs | 35,675 | 26.0% | 129,013 | 21.2% | 863,380 | 24.7% |
| 24-48hrs | 43,393 | 31.7% | 198,772 | 32.7% | 1,468,361 | 42.0% |
| >48hrs | 58,030 | 42.3% | 280,085 | 46.1% | 1,161,535 | 33.3% |

# Table D in S1 Text. Top source and sink municipalities of malaria importation by node type. Types were defined in Methods and Fig. 4b. Type D is the most common and the table includes only the top 15 municipalities.

| **SINK** | | | | |
| --- | --- | --- | --- | --- |
| Rank  (By Strength) | **Type A** | **Type B** | **Type C** | **Type D** |
| 1 | Manaus | Jaru | Buritis | Mâncio Lima |
| 2 | Boa Vista | Breves | Tailândia | Rodrigues Alves |
| 3 | Porto Velho | Belém | Itaituba | Careiro |
| 4 | Cruzeiro do Sul | Cacoal | Tefé | Laranjal do Jari |
| 5 | Macapá | Santarém | Boca do Acre | Benjamin Constant |
| 6 | Ariquemes | Manacapuru | Novo Progresso | Altamira |
| 7 | Tucuruí | Ouro Preto do Oeste | Anajás | Jacundá |
| 8 | Santana | Ananindeua | Afuá | Paragominas |
| 9 | Rio Branco |  |  | Porto Grande |
| 10 | Ji-Paraná |  |  | Jacareacanga |
| 11 | Humaitá |  |  | Machadinho D'Oeste |
| 12 |  |  |  | Anapu |
| 13 |  |  |  | Maués |
| 14 |  |  |  | Apuí |
| 15 |  |  |  | Oeiras do Pará |
| **Source** | | | | |
| Rank  (By Strength) | **Type A** | **Type B** | **Type C** | **Type D** |
| 1 | Porto Velho | Manaus | Canutama | Guajará |
| 2 | Itaituba | Anajás | Rodrigues Alves | Rio Preto da Eva |
| 3 | Pacajá | Colniza | Cantá | Moju |
| 4 | Candeias do Jamari | Machadinho D'Oeste | Lábrea | Maués |
| 5 | Cruzeiro do Sul | Humaitá | Careiro | Mazagão |
| 6 |  | Ariquemes | Alto Alegre | Autazes |
| 7 |  | Macapá | Iranduba | Porto Grande |
| 8 |  | Buritis | Nova Manoré | Presidente Figueiredo |
| 9 |  |  |  | Manicoré |
| 10 |  |  |  | Alvarães |
| 11 |  |  |  | Goianésia do Pará |
| 12 |  |  |  | Mucajaí |
| 13 |  |  |  | Itapuã do Oeste |
| 14 |  |  |  | Mâncio Lima |
| 15 |  |  |  | Pedra Branca do Amapari |

# Table E in S1 Text. Annual Pearson correlation between the Gross malaria Case Migration Rate (GCMR) and the Annual Parasite Index (API) for all municipalities in Brazil. LCI = Lower bound of 95% confidence interval. UCI = Upper bound of 95% confidence interval.

| **Year** | **Pearson Correlation** | **LCI** | **UCI** | **P-value** |
| --- | --- | --- | --- | --- |
| 2004 | 0.887 | 0.877 | 0.748 | <0.001 |
| 2005 | 0.888 | 0.895 | 0.771 | <0.001 |
| 2006 | 0.858 | 0.879 | 0.803 | <0.001 |
| 2007 | 0.813 | 0.897 | 0.794 | <0.001 |
| 2008 | 0.806 | 0.847 | 0.823 | <0.001 |
| 2009 | 0.808 | 0.869 | 0.802 | <0.001 |
| 2010 | 0.818 | 0.798 | 0.830 | <0.001 |
| 2011 | 0.781 | 0.827 | 0.743 | <0.001 |
| 2012 | 0.733 | 0.791 | 0.779 | <0.001 |
| 2013 | 0.729 | 0.820 | 0.771 | <0.001 |
| 2014 | 0.788 | 0.793 | 0.803 | <0.001 |
| 2015 | 0.809 | 0.822 | 0.762 | <0.001 |
| 2016 | 0.816 | 0.803 | 0.795 | <0.001 |
| 2017 | 0.762 | 0.831 | 0.857 | <0.001 |
| 2018 | 0.788 | 0.764 | 0.877 | <0.001 |
| 2019 | 0.779 | 0.796 | 0.823 | <0.001 |
| 2020 | 0.867 | 0.713 | 0.848 | <0.001 |
| 2021 | 0.836 | 0.752 | 0.809 | <0.002 |
| 2022 | 0.823 | 0.709 | 0.836 | <0.001 |
| All Years | 0.816 | 0.813 | 0.819 | <0.001 |

# Table F in S1 Text. Top and bottom 20 municipalities for mean Gross malaria Case Migration Rate (GCMR) and mean Annual Parasite Index (API), Brazil 2004-2022.

| Rank | **Bottom 20** | | | **Top 20** | | |
| --- | --- | --- | --- | --- | --- | --- |
|  | Municipality | Mean annual GCMR | Mean API | Municipality | Mean annual GCMR | Mean API |
| 1 | Caucaia | 0.0001 | 0.0001 | Canutama | 109.07 | 133.15 |
| 2 | Parnamirim | 0.0002 | 0.0000 | Rodrigues Alves | 108.47 | 372.15 |
| 3 | Jaboatão dos Guararapes | 0.0002 | 0.0000 | Alto Alegre | 86.80 | 197.08 |
| 4 | Olinda | 0.0002 | 0.0000 | Cantá | 74.91 | 202.78 |
| 5 | Arapiraca | 0.0002 | 0.0000 | Guajará | 68.61 | 187.52 |
| 6 | Ribeirão das Neves | 0.0002 | 0.0000 | Candeias do Jamari | 66.87 | 194.22 |
| 7 | Itaboraí | 0.0002 | 0.0000 | Jacareacanga | 66.66 | 298.03 |
| 8 | Mesquita | 0.0002 | 0.0001 | Itapuã do Oeste | 63.25 | 153.50 |
| 9 | São Gonçalo | 0.0002 | 0.0000 | Mâncio Lima | 60.34 | 404.30 |
| 10 | Araraquara | 0.0002 | 0.0000 | Pedra Branca do Amapari | 56.04 | 143.70 |
| 11 | Embu das Artes | 0.0002 | 0.0002 | Porto Grande | 55.91 | 111.66 |
| 12 | Francisco Morato | 0.0002 | 0.0000 | Serra do Navio | 54.08 | 133.69 |
| 13 | Guarulhos | 0.0002 | 0.0000 | Mucajaí | 54.06 | 92.20 |
| 14 | Itaquaquecetuba | 0.0003 | 0.0000 | Anajás | 50.21 | 378.46 |
| 15 | Jundiaí | 0.0003 | 0.0000 | Ferreira Gomes | 48.21 | 57.66 |
| 16 | Limeira | 0.0003 | 0.0000 | Alvarães | 47.64 | 135.40 |
| 17 | Osasco | 0.0003 | 0.0000 | Nova Mamoré | 46.00 | 90.97 |
| 18 | São Carlos | 0.0003 | 0.0002 | Careiro | 45.13 | 122.62 |
| 19 | São Vicente | 0.0003 | 0.0000 | Buritis | 43.75 | 32.22 |
| 20 | Sumaré | 0.0003 | 0.0000 | Mazagão | 43.04 | 129.44 |

# Table G in S1 Text. Spatio-temporal analysis of within- and between-state sources and sinks of malaria-infected individuals. Clustering was assessed with the Scan Statistic and only clusters with P<0.05 were included in the table (Methods).

| **Year** | **Source** | | | **Sink** | | |
| --- | --- | --- | --- | --- | --- | --- |
|  | **# of Clusters** | **Average radius of Clusters (Km)** | **Average Cluster Duration (Months)** | **# of Clusters** | **Average radius of Clusters (Km)** | **Average Cluster Duration (Months)** |
| **Within-State** | | | | | | |
| 2004 | 7 | 1000.30 | 4.88 | 8 | 740.69 | 4.76 |
| 2005 | 12 | 848.19 | 4.44 | 7 | 900.82 | 4.80 |
| 2006 | 5 | 1009.68 | 4.96 | 5 | 980.50 | 4.94 |
| 2007 | 6 | 1319.73 | 4.99 | 5 | 741.05 | 4.98 |
| 2008 | 2 | 1449.71 | 5.00 | 2 | 1112.85 | 5.00 |
| 2009 | 4 | 1022.51 | 3.27 | 3 | 1240.19 | 4.98 |
| 2010 | 3 | 1080.38 | 4.98 | 2 | 605.36 | 5.00 |
| 2011 | 2 | 700.10 | 5.00 | 2 | 600.87 | 4.59 |
| 2012 | 4 | 1330.32 | 4.97 | 4 | 960.30 | 2.57 |
| 2013 | 3 | 1266.95 | 4.31 | 3 | 1362.62 | 4.85 |
| 2014 | 5 | 1133.24 | 4.60 | 4 | 1269.20 | 4.84 |
| 2015 | 6 | 808.76 | 4.98 | 5 | 1223.86 | 5.00 |
| 2016 | 4 | 690.35 | 4.95 | 4 | 927.87 | 5.00 |
| 2017 | 5 | 1109.29 | 4.20 | 2 | 1142.17 | 5.00 |
| 2018 | 3 | 1176.52 | 5.00 | 3 | 1176.52 | 5.00 |
| 2019 | 2 | 1397.01 | 5.00 | 1 | 1407.06 | 5.00 |
| 2020 | 3 | 1301.81 | 4.99 | 5 | 837.09 | 4.97 |
| 2021 | 6 | 1157.32 | 4.82 | 4 | 902.07 | 4.78 |
| 2022 | 4 | 1327.32 | 5.00 | 5 | 918.48 | 4.99 |
| **Between-State** | | | | | | |
| 2004 | 4 | 488.46 | 5.00 | 7 | 749.50 | 4.99 |
| 2005 | 4 | 1212.71 | 5.00 | 8 | 742.62 | 4.94 |
| 2006 | 4 | 626.97 | 5.00 | 4 | 756.19 | 5.00 |
| 2007 | 4 | 793.71 | 4.67 | 10 | 609.26 | 3.14 |
| 2008 | 4 | 1186.75 | 4.88 | 5 | 1023.23 | 3.06 |
| 2009 | 5 | 816.35 | 3.33 | 4 | 1332.71 | 4.79 |
| 2010 | 3 | 1367.32 | 5.00 | 6 | 956.44 | 4.95 |
| 2011 | 3 | 1383.65 | 4.89 | 4 | 932.46 | 5.00 |
| 2012 | 5 | 879.39 | 4.93 | 4 | 796.94 | 4.58 |
| 2013 | 3 | 867.60 | 4.53 | 5 | 853.97 | 5.00 |
| 2014 | 2 | 1164.75 | 5.00 | 3 | 867.42 | 4.18 |
| 2015 | 2 | 1164.75 | 5.00 | 3 | 621.92 | 4.97 |
| 2016 | 3 | 979.80 | 3.85 | 6 | 1120.69 | 4.70 |
| 2017 | 3 | 1255.13 | 4.98 | 5 | 886.33 | 4.92 |
| 2018 | 3 | 1215.38 | 5.00 | 5 | 912.99 | 4.48 |
| 2019 | 2 | 994.18 | 5.00 | 5 | 434.20 | 4.99 |
| 2020 | 4 | 986.49 | 4.96 | 5 | 436.54 | 4.92 |
| 2021 | 4 | 391.14 | 4.91 | 4 | 392.78 | 5.00 |
| 2022 | 3 | 855.81 | 5.00 | 4 | 380.35 | 4.90 |

# Table H in S1 Text. Clustering analysis of Gross Case Migration Rate (GCMR) of infected individuals, using the Scan Statistics. Only clusters with P<0.05 were included (Methods).

| **Year** | **Within-State GCMR** | | | **Between-State GCMR** | | | **Total GCMR** | | |
| --- | --- | --- | --- | --- | --- | --- | --- | --- | --- |
|  | **# of Clusters** | **Average radius of Clusters (Km)** | **Average Cluster Duration (Months)** | **# of Clusters** | **Average radius of Clusters (Km)** | **Average Cluster Duration (Months)** | **# of Clusters** | **Average radius of Clusters (Km)** | **Average Cluster Duration (Months)** |
| 2004 | 15 | 119.61 | 3.70 | 8 | 159.12 | 4.92 | 17 | 102.58 | 3.53 |
| 2005 | 16 | 124.15 | 2.29 | 7 | 61.76 | 4.40 | 19 | 111.76 | 1.67 |
| 2006 | 14 | 116.08 | 4.06 | 6 | 51.03 | 4.90 | 19 | 148.11 | 3.06 |
| 2007 | 16 | 157.80 | 3.58 | 9 | 106.16 | 3.89 | 17 | 136.82 | 3.52 |
| 2008 | 22 | 76.58 | 2.46 | 9 | 97.44 | 2.20 | 25 | 101.07 | 2.89 |
| 2009 | 15 | 115.04 | 1.49 | 13 | 148.43 | 4.68 | 16 | 162.03 | 1.31 |
| 2010 | 12 | 18.08 | 3.57 | 13 | 89.60 | 3.57 | 11 | 56.55 | 4.07 |
| 2011 | 9 | 76.73 | 2.24 | 5 | 0.00 | 4.20 | 11 | 101.71 | 2.23 |
| 2012 | 15 | 75.29 | 2.72 | 7 | 202.55 | 4.91 | 15 | 70.93 | 2.80 |
| 2013 | 14 | 30.16 | 2.71 | 6 | 89.93 | 4.11 | 16 | 26.98 | 2.42 |
| 2014 | 10 | 66.15 | 3.38 | 6 | 132.73 | 3.75 | 11 | 62.25 | 3.18 |
| 2015 | 7 | 83.40 | 1.88 | 7 | 349.51 | 3.05 | 8 | 78.50 | 1.94 |
| 2016 | 2 | 132.29 | 3.13 | 3 | 55.71 | 5.00 | 4 | 105.83 | 2.90 |
| 2017 | 16 | 77.13 | 1.48 | 5 | 113.26 | 4.67 | 16 | 33.61 | 2.15 |
| 2018 | 8 | 106.85 | 3.50 | 4 | 114.64 | 2.75 | 11 | 60.30 | 3.38 |
| 2019 | 10 | 72.11 | 4.00 | 6 | 208.44 | 3.86 | 11 | 68.32 | 2.47 |
| 2020 | 5 | 43.53 | 2.57 | 5 | 0.00 | 4.60 | 6 | 38.09 | 3.00 |
| 2021 | 1 | 0.00 | 4.00 | 2 | 0.00 | 4.50 | 2 | 0.00 | 2.50 |
| 2022 | 1 | 0.00 | 4.00 | 3 | 0.00 | 4.67 | 1 | 0.00 | 4.00 |

# Figure A in S1 Text. Annual Parasite Index, Brazil 2004-2022. Calculated as the number of confirmed malaria cases in the municipality of infection divided by the population of the municipality multiplied by 1,000. Shapefile source: https://www.ibge.gov.br/en/geosciences/territorial-organization/territorial-meshes/18890-municipal-mesh.html?edicao=33161&t=o-que-e.

**
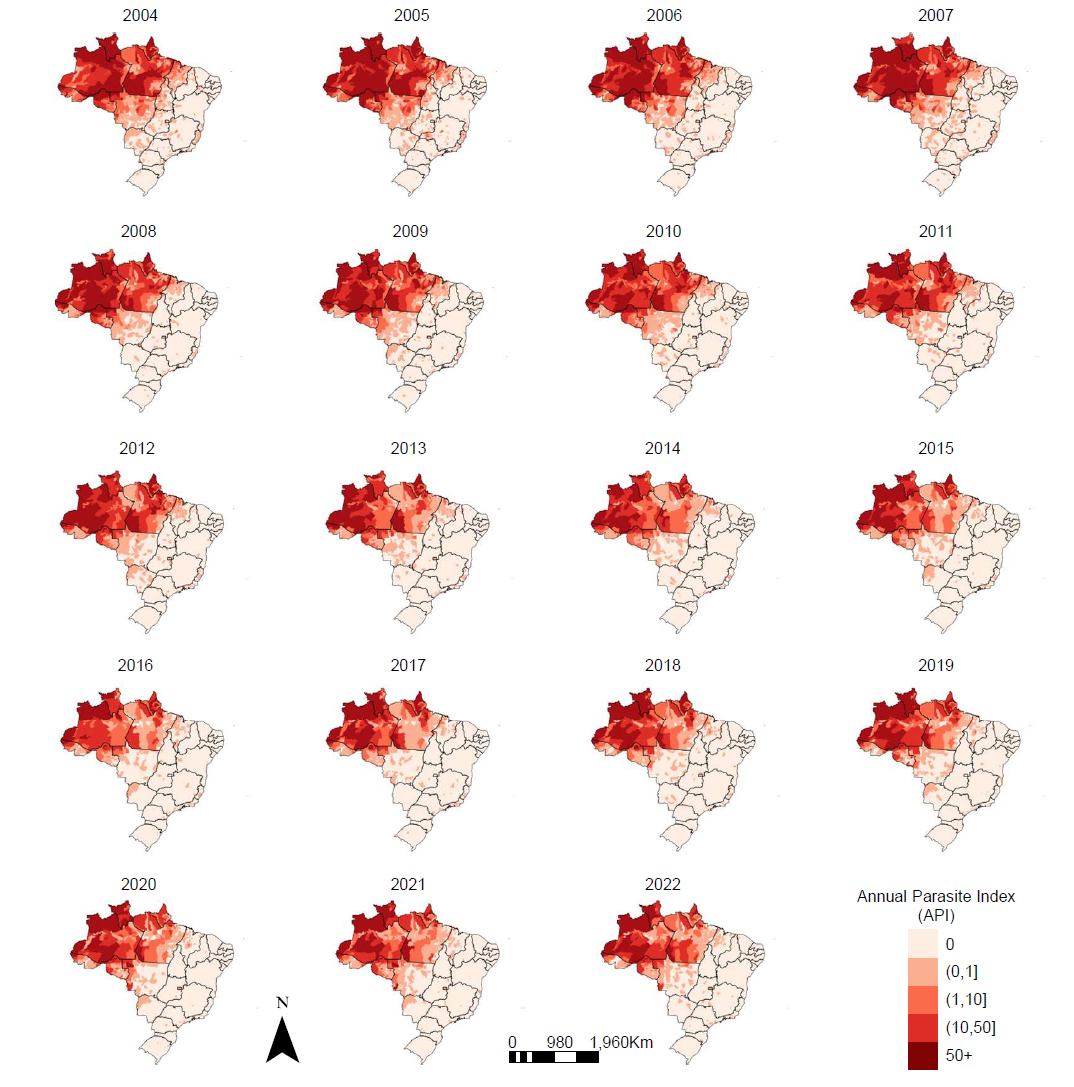
**

#

# Figure B in S1 Text. Seasonal multiplicative pattern of locally acquired and imported malaria cases by month, Brazil 2004-2022. The seasonal multiplicative component was obtained by time series decomposition, removing trend and random noise (Methods). State acronyms: AC=Acre, AP=Amapá, AM=Amazonas, PA=Pará, RO=Rondônia, RR=Roraima, TO=Tocantins, MA=Maranhão, and MT=Mato Grosso.

**
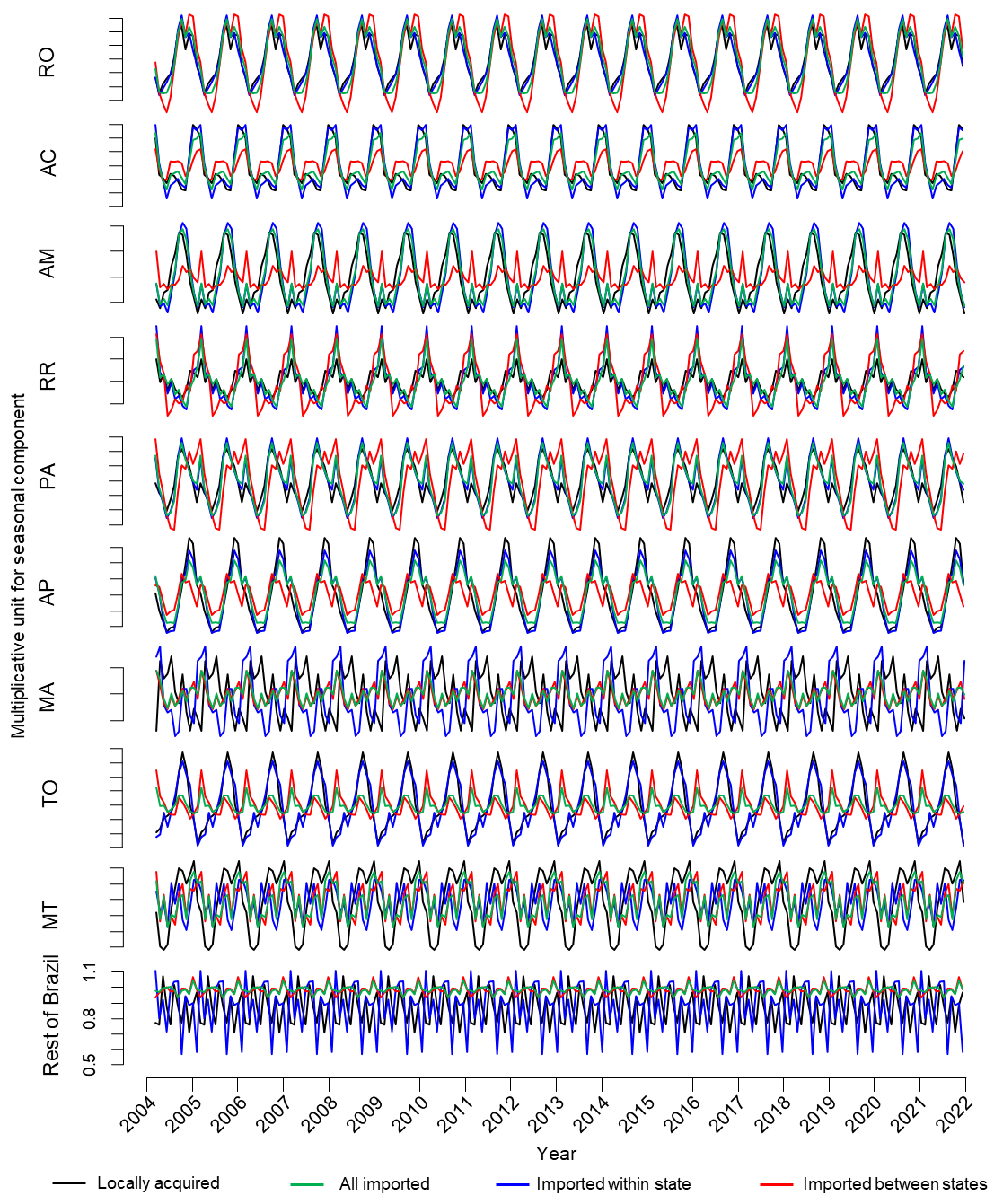
**

# Figure C in S1 Text. Boxplot of the coefficient of variation for each mobility flow. Calculation based on monthly municipal data for the years 2004 to 2022.


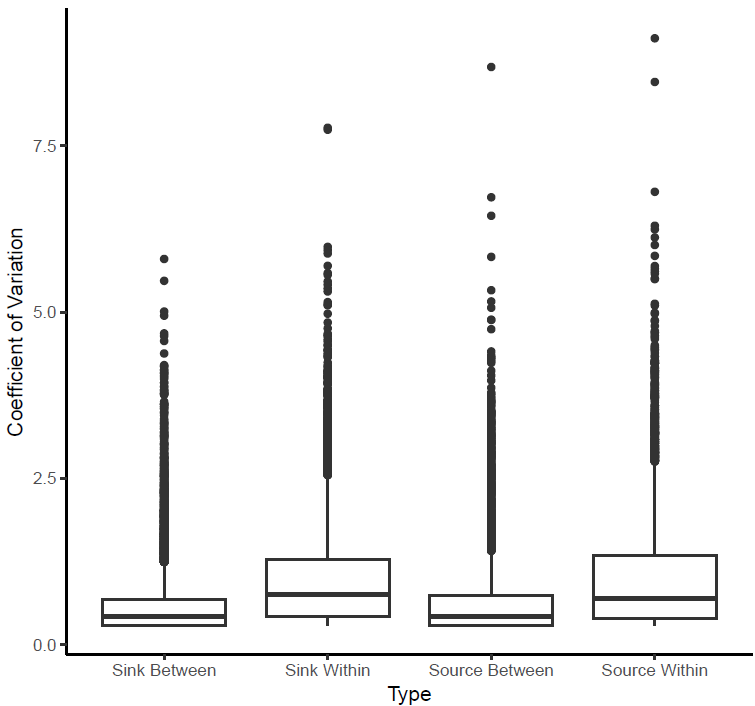


# Figure D in S1 Text. Malaria by period, Brazil 2004-2022. (Top) Boxplots of the Annual Parasite Index considering municipalities that were part of significant spatiotemporal clusters, by cluster type and period. (Bottom) Regression lines and R2 between the ratio relating imported and locally acquired cases (RILA) and API, by state and period. The state of Tocantins has a large confidence interval in 2017-2022 because it reported very few malaria cases during that period.


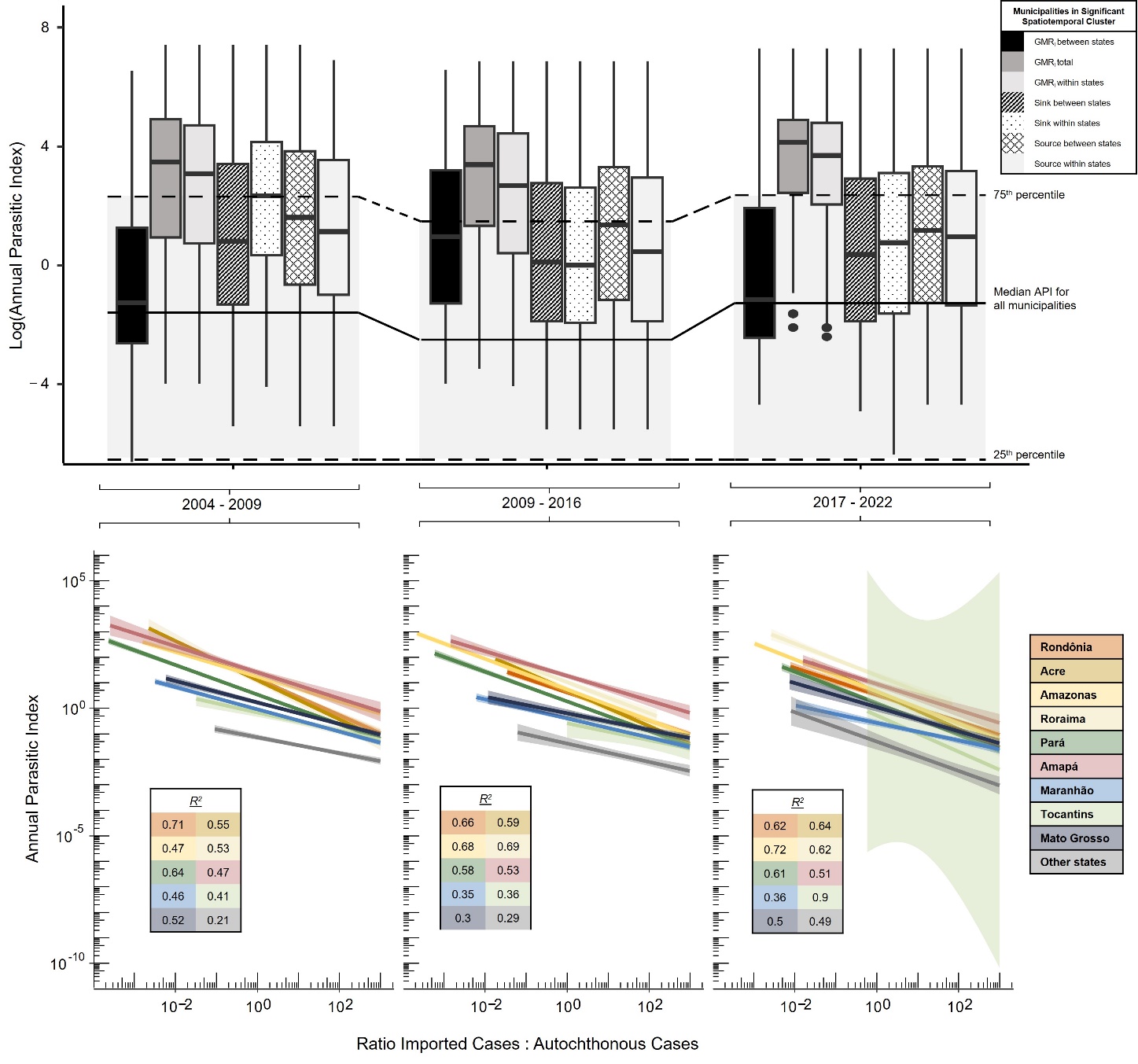


# Figure E in S1 Text. Correlation between the Gross malaria Case Migration Rate (GCMR) and the Annual Parasite Index (API). All Brazilian municipalities and years 2004-2022


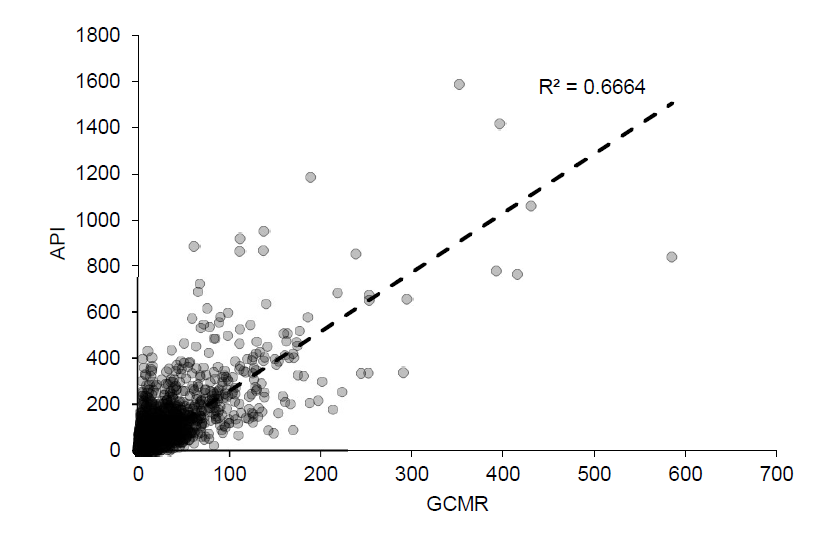

Supplement: S1 Text — Table A. Total and imported malaria cases (only between/within-states in Brazil), detailed by within- and between-state movements for all of Brazil, 2004–2022. Table B. Top 10 most common malaria importation routes in Brazilian municipalities considering the years 2004 to 2022. State acronyms: AC = Acre, AM = Amazonas, PA = Pará, RO = Rondônia, RR = Roraima, and MT = Mato Grosso. Table C. Summary groupings of Sivep-Malaria. The analysis includes only states in the Brazilian Amazon (Acre, Rondônia, Amazonas, Roraima, Pará, Amapá, Tocantins, Maranhão, and Mato Grosso) for the years 2004 to 2022. Table D. Top source and sink municipalities of malaria importation by node type. Types were defined in Methods and Fig 4B. Type D is the most common and the table includes only the top 15 municipalities. Table E. Annual Pearson correlation between the Gross malaria Case Migration Rate (GCMR) and the Annual Parasite Index (API) for all municipalities in Brazil. LCI = Lower bound of 95% confidence interval. UCI = Upper bound of 95% confidence interval. Table F. Top and bottom 20 municipalities for mean Gross malaria Case Migration Rate (GCMR) and mean Annual Parasite Index (API), Brazil 2004–2022. Table G. Spatio-temporal analysis of within- and between-state sources and sinks of malaria-infected individuals. Clustering was assessed with the Scan Statistic and only clusters with P<0.0006 were included in the table (Methods). Table H. Clustering analysis of Gross Case Migration Rate (GCMR) of infected individuals, using the Scan Statistics. Only clusters with P≤0.001 were included (Methods). Fig A. Annual Parasite Index, Brazil 2004–2022. Calculated as the number of confirmed malaria cases in the municipality of infection divided by the population of the municipality multiplied by 1,000. Shapefile source: https://www.ibge.gov.br/en/geosciences/territorial-organization/territorial-meshes/18890-municipal-mesh.html?edicao=33161&t=o-que-e. Fig B. Seasonal multiplicative pattern of lo [file pgph.0003452.s001.docx]
